# Advances in Genetics of Regeneration in Metabesity

**DOI:** 10.3390/genes10050383

**Published:** 2019-05-20

**Authors:** Benoit R. Gauthier, Francisco J. Bermúdez-Silva

**Affiliations:** 1 Andalusian Center of Molecular Biology and Regenerative Medicine-CABIMER, Junta de Andalucia-University of Pablo de Olavide-University of Seville-CSIC, 41092 Seville, Spain; 2 Centro de Investigación Biomédica en Red de Diabetes y Enfermedades Metabólicas Asociadas (CIBERDEM), 28029 Madrid, Spain; 3 Instituto de Investigación Biomédica de Málaga-IBIMA, UGC Endocrinología y Nutrición, Hospital Regional Universitario de Málaga, 29009 Málaga, Spain

**Keywords:** regeneration, metabolic diseases, genetic networks, genes, transgenic, animal model, inflammation, regenerative medicine, cell therapy

## Abstract

‘Metabesity’ is a recent term comprising a wide range of diseases with underlying metabolic disarrangements at its root, and whose aetiology lies in complex relationships among genes and the obesogenic environment to which individuals are currently exposed in most countries. Of note, epigenetic changes are increasingly being reported to play an outstanding role in carrying deleterious information that, together with susceptibility genes, boost the development of metabesity in subsequent generations. In this context, it is noteworthy to mention that the transition from the pre-industrial era to the current high-technology society and global economy, even after suffering two world wars, has been very fast. By contrast, evolution-driven processes, such as biological ones, are slow. In fact, there is a general consensus that at the metabolic level, adipogenic processes and thrifty pathways prevail over those promoting energy expenditure in a way that currently leads to metabolic diseases by excessive energy storage. In such an imbalanced social–biological scenario, genes that were beneficial in the past have shifted to becoming detrimental, i.e., favouring metabesity, which is quickly growing to reach pandemic proportions.

Metabesity includes but is not limited to obesity (currently not considered a disease in most countries), cardiovascular disease, diabetes and metabolic syndrome. From a pathophysiological point of view, its progression comprises inflammatory and oxidative damage, insensitivity to key regulators, e.g., hormones like insulin and leptin, cell death and overload of the natural regeneration capacity of tissues. While different approaches aiming at tackling metabesity are being explored, regenerative medicine remains a promising one, though facing important obstacles.

This Special Issue contains a series of new reviews and original research articles providing advances in this exciting field, highlighting some genes and processes that show potential in contributing to the development of new approaches for tackling metabesity. One such process is epigenetic regulation which, in the context of pancreatic regeneration in diabetes, is concisely but at the same time accurately reviewed by Dr. Collombat and co-workers [1]. Current research on this hot topic is unravelling a crucial role of epigenetics and epigenetic regulators during pancreas development; interestingly, the epigenetic signature is modified in obesity and T2D [1]. Of note, this kind of alteration not only induces a loss of identity and, consequently, function in beta and alpha cells but also seems to be present in adipocytes contributing to insulin resistance [1]. Indeed, epigenetic regulators and chromatin modifiers, such as lncRNAs and high mobility group (HMG) box-containing protein 20A (HMG20A), are emerging as key elements in maintaining the expression of transcription factors that are essential to beta-cell identity and function [1]. 

Regarding this latter factor, HMG20A, emerging evidence shows its involvement in neuronal and beta cell mature function [2,3]. It is noteworthy that blood glucose levels are maintained not only by the islet regulatory response but also through specific brain areas, like the hypothalamus and brain stem, where astrocytes seem to play an important role in glucosensing [4]. We contribute to this Special Issue by reviewing current knowledge on the brain–islet crosstalk that regulates and fine-tunes glucose homeostasis, and how specific cell types in both organs, especially beta cells and astrocytes, are damaged during metabesity development [4]. Indeed, given HMG20A regulates gene expression of key genes such as the basic helix–loop–helix transcription factor NEUROD, glucokinase (GK), and glucose transporters (GLUTs), common to astrocytes and beta cells, we hypothesise that HMG20A is a master regulator of glucose homeostasis whose targeting in metabesity could potentially lead to a dual beneficial effect on both islets and CNS, though more research is needed [4].

In addition, reflecting even more the current abundance of epigenetic studies in metabesity, Dr. Tinahones and collaborators here describe a differential methylation pattern of complement factor C3 in adipocytes from class 3 obese patients [5]. Accordingly, C3 methylation negatively correlates with BMI and leptin, while C3 mRNA shows a positive correlation with glucose, insulin and HOMA-IR [5]. Interestingly, complement factor C3 has recently been described to be highly expressed in human islets, protecting against beta cell loss by activating autophagy [6]. Thus, complement factor C3 arises as an attractive complement-derived molecule whose physiology and regulation during metabesity deserve more attention. 

Other interesting and emerging targets in metabesity are glucose regulated protein 78kD (GRP78), a chaperone known to reduce ER stress upstream of unfolded protein response (UPR) by improving protein folding. Dr. Lopez and co-workers show in this Special Issue that GRP78 overexpression in the ventromedial hypothalamus (a key brain area regulating thermogenesis) of rats fed a very high-fat diet decreases body weight, insulin resistance and hepatic steatosis, while increasing thermogenesis in brown adipose tissue and browning in white adipose tissue [7]. Although the clinical relevance of these findings is unknown, as the authors recognise, it is promising that chemical chaperones like tauroursodeoxycholic acid (TUDCA) or 4-phenyl butyric acid (4-PBA), very well tolerated in humans, have been approved by the United States Food and Drug Administration for the treatment of primary biliary cirrhosis and urea cycle disorders, respectively [8]. Therefore, in the context of metabesity, further research on the role of hypothalamic GRP78 is warranted. 

As can also be inferred from this Special Issue [4,7], the hypothalamus is a key structure in controlling energy homeostasis, and mutations in genes governing its physiological activity are direct causes of metabesity. Together with well-known examples like Leptin and MC4R, prohormone convertase subtilisin/kexin type 1 (PCSK1) is also a gene whose mutation leads to a phenotype of severe early-onset obesity. In this Special Issue, Dr. Creemers and co-workers review the contribution of PCSK1 to obesity physiopathology, taking advantage of recent and controversial studies suggesting a role of PCSK1 in Prader–Willi syndrome (PWS) (another clinical entity associated with severe obesity and sharing with PCSK1 deficiency several other symptoms, such as hypogonadotropic hypogonadism and growth retardation) [9]. The take-home message is that a deeper comprehension of the molecular mechanisms underlying the cellular actions of PC1/3 (the transcript product of PCSK1) could result in the identification of novel therapeutic targets of benefit not only to PWS and PC1/3-deficient patients but also in general obesity.
